# Optimization of protease production from surface-modified coffee pulp waste and corncobs using *Bacillus* sp. by SSF

**DOI:** 10.1007/s13205-016-0481-z

**Published:** 2016-08-12

**Authors:** Selvam Kandasamy, Govarthanan Muthusamy, Senthilkumar Balakrishnan, Senbagam Duraisamy, Selvankumar Thangasamy, Kamala-Kannan Seralathan, Sudhakar Chinnappan

**Affiliations:** 1 Centre for Biotechnology, Muthayammal College of Arts and Science, Rasipuram, Namakkal, Tamil Nadu 637 408 India; 2 Department of Applied Sciences, College of Environmental Technology, Muroran Institute of Technology, 27-1 Mizumoto, Muroran, Hokkaido 050-8585 Japan; 3 Department of Medical Microbiology, College of Health and Medical Sciences, Haramaya University, P.O. Box 235, Harar, Ethiopia; 4 Department of Marine Biotechnology, Bharathidasan University, Tiruchirappalli, Tamil Nadu 620 024 India; 5 PG and Research Department of Biotechnology, Mahendra Arts and Science College (Autonomous), Kalippatti, Namakkal, Tamil Nadu 637501 India; 6 Division of Biotechnology, Advanced Institute of Environment and Bioscience, College of Environmental and Bioresource Sciences, Chonbuk National University, Iksan, 570752 South Korea

**Keywords:** Protease, SSF, *Bacillus* sp., Coffee pulp waste, Corncobs, Response surface methodology

## Abstract

The aim of the study was to identify new sources of substrate from agro-industrial waste for protease production using *Bacillus* sp., a local bacteria isolated from an agro-waste dumping site. The strain was identified as *Bacillus* sp. BT MASC 3 by 16S rRNA sequence followed by phylogenic analysis. Response surface methodology-based Box–Behnken design (BBD) was used to optimize the variables such as pH, incubation time, coffee pulp waste (CPW) and corncob (CC) substrate concentration. The BBD design showed a reasonable adjustment of the quadratic model with the experimental data. Statistics-based contour and 3-D plots were generated to evaluate the changes in the response surface and understand the relationship between the culture conditions and the enzyme yield. The maximum yield of protease production (920 U/mL) was achieved after 60 h of incubation with 3.0 g/L of CPW and 2.0 g/L of CC at pH 8 and temperature 37 °C in this study. The molecular mass of the purified enzyme was 46 kDa. The highest activity was obtained at 50 °C and pH 9 for the purified enzymes.

## Introduction

Proteases (EC 3.4.21–24) are a cluster of enzyme which hydrolyzes proteins, that is, catabolizes proteins by hydrolysis of the peptide bonds that link amino acids together in the polypeptide chain forming the proteins. Proteases also known as peptidyl-peptide hydrolase and constitute 60–65 % of the global enzyme market (Shankar et al. 2011). Proteases are commercially important enzymes having a wide range of applications in various industrial, biotechnological, medicinal and basic research fields (Das and Prasad 2010).

Proteases are widely distributed in the microbial population, viz. bacteria, actinomycetes and fungi. Although proteases are widespread in nature, microbes serve as a preferred source of these enzymes and account for around two-thirds of commercial production worldwide. Though proteases are produced by a variety of bacteria such as *Pseudomonas aeruginosa*, *Flavobacterium*, *Clostridium*, *Achromobacter*, *Thermo actinomyces* and species belonging to *Streptomyces*, *Bacillus* sp. is the major source which secretes a variety of soluble extracellular enzymes (Selvam et al. 2016; Jellouli et al. 2008; Ellaiah et al. 2002). These strains are specific producers of extracellular proteases and can be cultivated under extreme temperature (40–60 °C) and pH (9–11) conditions to give rise to products that are, in turn, stable in a wide range of harsh environments (Eichler 2001; Han and Damodaran 1997). Currently, many economically important industrial enzymes are produced by cultivation of bacteria, such as *Bacillus* sp. (Hoa et al. 2000).

Solid-state fermentation (SSF) has been defined as the fermentation process which involves solid matrix and is carried out in the absence or near absence of free water; however, the substrate must possess enough moisture to support growth and metabolism of the microorganism. The solid matrix could be either the source of nutrients or simply a support impregnated by the proper nutrients that allows the development of the microorganisms (Pandey 2003). Recently, SSF has generated much interest because of lower manufacturing costs by utilizing unprocessed or moderately processed raw materials. SSF is generally a simpler process and requires less pre-processing energy than submerged fermentation. Other advantages are superior productivity, low waste water output and improved product recovery. It therefore becomes very important to determine the environmental conditions of the microorganism for maximum production (Pandey et al. 2000). However, several bacterial enzymes, such as alpha amylase, cutinase, cellulase, protease, xylanase and keratinase have been successfully produced in SSF using a wide range of bacteria (Dhillon et al. 2012).

Response surface methodology (RSM) is now being routinely used for optimization studies in several biotechnological and industrial processes (Beg et al. 2003). A statistically designed optimization study is helpful in confirming previous effects and interactions of fermentation variables and in determining the optimum values of the critical factors (He et al. 2004). The application of statistical experimental design techniques in fermentation process development can result in an improvement of product yield, reduce process variability, give closer confirmation of the output response to the experimental values and reduce overall costs. RSM can be used to evaluate the relative significance of several factors even in the presence of complex interactions (Selvam et al. 2014; Pansuriya and Singhal 2010).

The selection of an ideal agro-biotech waste for enzyme production in a solid-state fermentation process depends upon several factors, mainly related to the cost and availability of the substrate material, and thus may involve screening of several agro-industrial residues. Coffee pulp wastes (CPW) are generated during the industrial processing of coffee cherries by wet and/or dry process. These wastes are generated by coffee-producing countries (India, Brazil, Vietnam, etc.) in large amount throughout the year and are the most abundant renewable resources (Pandey et al. 2000, Roussos et al. 1995). Corncobs (CC), the central part of maize (*Zea mays*), are either thrown out as waste or burnt, an application with low added value, causing environmental concern. So it is an exciting research area to use corncob for chemical processing to obtain end products with added values worldwide at a very low price (Kumar et al. 2010).

The objectives of this study were as follows: (1) identification and screening of protease-producing bacteria from agro-waste dumping site, (2) evaluation of the potential of the agro-industrial wastes, CPW and CC as a substrates for protease production under SSF, and (3) optimization of the physicochemical condition by BBD.

## Materials and methods

### Isolation and screening of protease-producing bacteria

The soil sample was collected from the agricultural waste dumping area in Salem, Tamil Nadu, India. The bacteria were isolated from the soil samples according to Kamala-Kannan and Krishnamoorthy (2006) with minor modifications. 1 g of soil sample was taken in a 250 mL conical flask containing 100 mL of sterile distilled water and the contents were mixed well in an orbital shaker to get a homogeneous suspension. The suspension was serially diluted 10^7^ times and using pour plate technique the diluted samples were transferred to Petri dishes containing sterile nutrient agar medium. After inoculation, the plates were incubated at 37 °C for 48 h. After incubation, the isolated colonies were identified.

The isolated bacterial culture was inoculated on casein agar medium containing casein 2.0 %; peptone 0.5 % and agar 1.5 % and then incubated at 37 °C for 24 h and observed in the clear halo zone around the colonies. Depending on the zone of clearance, the strain of BT MASC 3 was selected for further experimental studies.

## 16S rRNA amplification and partial gene sequencing of the bacterial isolate BT MASC 3

Genomic DNA was extracted according to Sambrook and Russell (2001). The partial 16S rRNA gene was amplified using universal primers 27f (5′AGAGTTTGATCCTGGCTCAG3′) and 907r (5′CCCCGTCAATTCATTTGAGTTT3′). The amplicon was purified (QIAGEN, CA, USA) and sequenced using ABI PRISM (Model 3700, CA, and USA). The sequences were compared using BLAST (NCBI) for the identification of bacterial isolate BT MASC 3. Phylogenetic analysis was performed using the neighbor-joining method in the CLC WORKBENCH 6 software (CLC bio, MA, USA).

### Substrate

The CPW was procured from the coffee-processing industry at Yercaud, Salem, Tamil Nadu, India. CC was collected from the local agricultural field, Salem, Tamil Nadu, India, and dried in a hot air oven for 10 min at 100 °C. Then the husk was ground in a blender to prepare particles with a mean size of 1.0–2.0 mm. The alkali pre-treatment of substrates was conducted with 1:10 ratio using 1 % (w/v) NaOH (1 g of substrate in 10 mL of 1 % NaOH solution) (Wang et al. 2004). Alkali pre-treated substrates were then washed with distilled water and neutralized to around pH 7 and base followed by drying (Bansal et al. 2011).

### Scanning electron microscopy

Scanning electron microscopy (Jeol JSM 6390 model) was used to examine the morphological modifications of CPW and CC before and after the alkaline pre-treatment according to Díaz-Malváez et al. (2013) Samples were dehydrated and mounted on stubs and sputter-coated with gold for 300 s using high vacuum and a voltage acceleration of 10 kV.

### Characterization of alkali-treated substrates

FTIR spectroscopy was used as an analytical tool to qualitatively determine the chemical changes in the lignocellulosic material upon pre-treatment. FTIR spectra of untreated and alkali-treated substrates were obtained by direct transmittance using the KBr pellet technique (Shimadzu). The spectra of 400–4000 cm^−1^ were used at a spectral resolution of 1 cm^−1^ (Sun et al. 2007).

### Solid-state fermentation

Five grams of CPW and CC was put in Erlenmeyer flasks and moistened (60–70 %) with ultrapure water. The contents were vigorously mixed, and the flasks were autoclaved at 121 °C for 15 min. After sterilization, the flasks were cooled to 50 °C and inoculated with 3 mL of BT MASC 3 isolate carrying 10^8^ cells/mL (0.8 OD at 600 nm) as a seed culture under aseptic condition. The contents of the flasks were well mixed and incubated at 36 ± 1 °C for 96 h.

### Statistical optimization of protease production

The experiment is a well accepted statistical technique able to design and optimize the experimental process that involves choosing the optimal experimental design and estimate the effect of the several variables independently and also the interactions simultaneously. Response surface methodology combined with BBD was established using Design Expert software (9.0.0.7 trial version). Four factors, namely, pH, incubation time, CPW and CC, were optimized for enhanced protease production using the isolate BT MASC 3 under SSF. Based on BBD, the factors were analyzed at two levels: −1, for low level, and +1, for high level. A total of 29 runs were performed to optimize the process parameters, and experiments were performed according to the experimental design matrix. The results were evaluated by applying the coefficient of determination (*R* ^2^), analysis of variance (ANOVA) and response plots. Employing RSM, the most widely used second-order polynomial equation was developed to fit the experimental results and identify the relevant model terms:

1 $$Y \, = \beta_{0} \varSigma \beta_{i} X_{i} \, + \, \varSigma \beta_{i} X_{i} \beta_{ij} \, + \, \varSigma \, X_{i} X_{j} ,$$

where *Y* is the predicted response; *β*_0_, *β*_*i*_, and *β*_*ij*_ are constant regression coefficients of the model; and *X*_*i*_ and *X*_*j*_ represent independent variables. The experimental design chosen for the study was a Box–Behnken design that helps in investigating linear, quadratic and cross product effects of these factors, each varied at these levels, and also includes three center points for replication (Govarthanan et al. 2015; Ellaiah et al. 2002).

### Enzyme recovery and purification

The enzyme was extracted after the incubation period from the fermentation medium by mixing thoroughly with 50 mM glycine–NaOH buffer, pH 11, for 30 min, and the extract was separated by filtering initially through Whatman No. 1 filter paper followed by 0.2 μm membrane filter and centrifuged at 6000 rpm for 10 min. The supernatant was subjected to 65 % ammonium sulfate saturation. After that, the solution was centrifuged at 10,000 rpm, 4 °C for 30 min, and the precipitate containing the enzyme was collected. The pellet was re-dissolved in 0.2 M phosphate buffer (pH 7.0). Then, the suspension was dialyzed thoroughly against the same buffer for desalting. The purified enzyme was used for protease assay (Prakasham et al. 2006).

### Enzyme assay

Protease activity was estimated by the Anson–Hagihara method (Patel et al. 2006). The enzyme (0.5 mL) was added to 3.0 mL casein (0.6 % in 20 mM glycine–NaOH buffer, pH 10) and the reaction mixture was incubated at 37 °C for 10 min before the addition of 3.2 mL of M trichloroacetic acid (TCA mixture 0.11 M TCA, 0.22 M sodium acetate, 0.33 M acetic acid). The terminated reaction mixture was incubated for 30 min at 30 °C temperature. The precipitates were removed by filtration through Whatman No. 1 filter paper and the absorbance of the filtrate was measured at 280 nm. One unit of protease activity was defined as the amount of enzyme liberating 1 μg of tyrosine per minute under the assay conditions. Enzyme units were measured using tyrosine (0–100 μg) as a standard (Satbir and Bijender 2016; Govarthanan et al. 2014).

### Determination of protein concentration

The protein concentration of the crude enzyme as well as the purified enzyme was determined by the method of Lowry et al. (1951) using bovine serum albumin as a standard.

### Determination of molecular weight of the protease enzyme using SDS-PAGE and zymogram

The molecular mass of the purified enzyme was determined on 12 % sodium dodecyl sulfate polyacrylamide gel electrophoresis (SDS-PAGE) according to the method of Leammli (1970). About 10 μg of the purified protein was loaded on 12 % SDS-PAGE with standard molecular mass markers (GENEI, Bangalore, India) and electrophoresis was carried out at a constant current of 30 mA. After electrophoresis, the gel was stained with Coomassie Brilliant Blue R-250 (CBB R-250) and destained to visualize protein bands over the UV-Transilluminator (Biotech, Yercaud, Salem, Tamil Nadu, India).

The zymogram was performed using 10 mg/mL gelatin in 10 % polyacrylamide mixture. The purified and concentrated sample of the enzyme was loaded without subjecting it to the heat treatment in the gel loading buffer devoid of *β*-mercaptoethanol. Electrophoresis was performed at a constant voltage of 80 V and 10 mA for 3–5 h. On completion, the gel was treated with Triton X-100 (2.5 % v/v) by soaking for 20 min with three repetitions of the process. The gel was incubated for 12 h in 50 mM glycine–NaOH buffer of pH 8.5 at room temperature. The gel was stained with Coomassie Brilliant Blue R-250 (0.5 %) and destained with methanol–glacial acetic acid–water (30:10:60). The digestion of the substrate is indicated by clear areas.

### Effect of pH and temperature on protease activity and stability

Optimum pH was determined by estimating the protease activity at 50 °C and pH values ranging from 6 to 11 (citrate, pH 6; phosphate, pH 7; Tris–HCl, pH 8; carbonate, pH 9 and 10; sodium phosphate–NaOH, pH 11). The stability of protease was examined by incubating the enzyme at 28 °C in buffers at pH values ranging from 6 to 11 for 1 h. Residual activity was estimated as described earlier and expressed as percentage of the initial activity taken as 100 %.

The optimum temperature was determined by estimating the protease activity at pH 9 and temperatures ranging from 30 to 70 °C for 10 min. The thermal stability was examined by incubating the enzyme at temperatures ranging from 30 to 70 °C for 1 h and the residual activity was measured at 50 °C, pH 9, and expressed as percentage of initial activity taken as 100 %.

## Results and discussion

### Isolation, identification, screening and characterization of protease-producing bacteria

This study represents an attempt to evaluate the potential of CPW, CC and bacteria from agricultural waste for the production of industrially important protease by SSF. The protease producing bacteria was isolated from waste dumping site and screened on skimmed milk agar plates for caseinolytic activity. The results showed that the isolate, designated BT MASC 3, exhibited maximum caseinolytic activity.

**Fig. 1:**
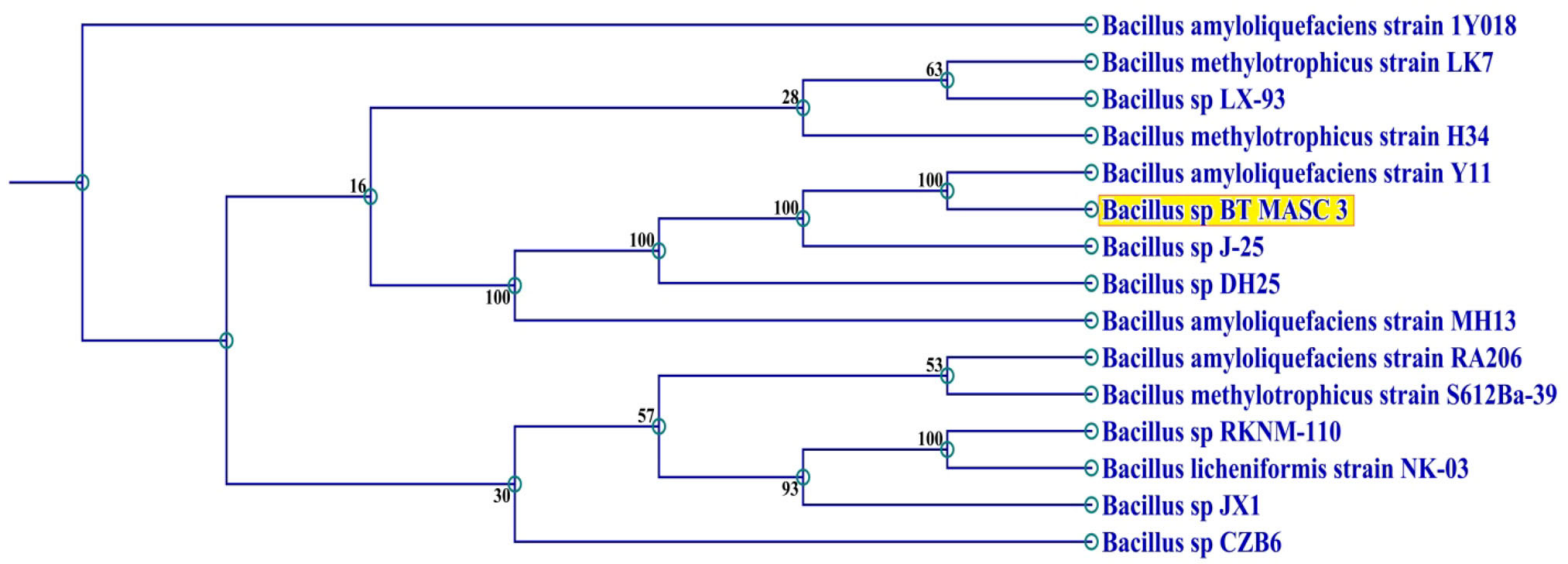
16S rRNA-based phylogenetic analysis of *Bacillus* sp. BT MASC 3. Bootstrap values and *scale bar* depicting the substitution rate per site are indicated. The phylogenetic tree constructed by the neighbor-joining method showing the position of isolate BT MASC 3

The isolate BT MASC 3 was selected for SSF studies. Polymerase chain reaction amplification of the partial 16S rRNA resulted in the predicted 907-bp amplicon in the isolate BT MASC. The amplicon sequences were compared with 16S rRNA sequences in the NCBI database, and the sequences exhibited 99 % identity with *Bacillus* sp. The partial 16S rRNA of the isolate BT MASC 3 was deposited in GenBank (Accession Number: KJ447124). The results are in accordance with several studies reporting the protease-producing *Bacillus* sp. under SSF (Prakasham et al. 2006). A phylogenetic tree was derived from the partial 16S rRNA sequences of the isolate BT MASC 3 with existing sequences in the database, and the results are shown in Fig. 1. The phylogenetic tree was constructed from the sequence data by the neighbor-joining method. Expectedly, the isolate BT MASC 3 and *Bacillus* sp. were in the same clusters and which further confirms the identity of the 16S rRNA sequence with *Bacillus* sp. The *Bacillus* species are considered to be the most important sources of protease and have been used for enzyme production using SSF (Prakasham et al. 2006).

### Scanning electron microscopy

**Fig. 2:**
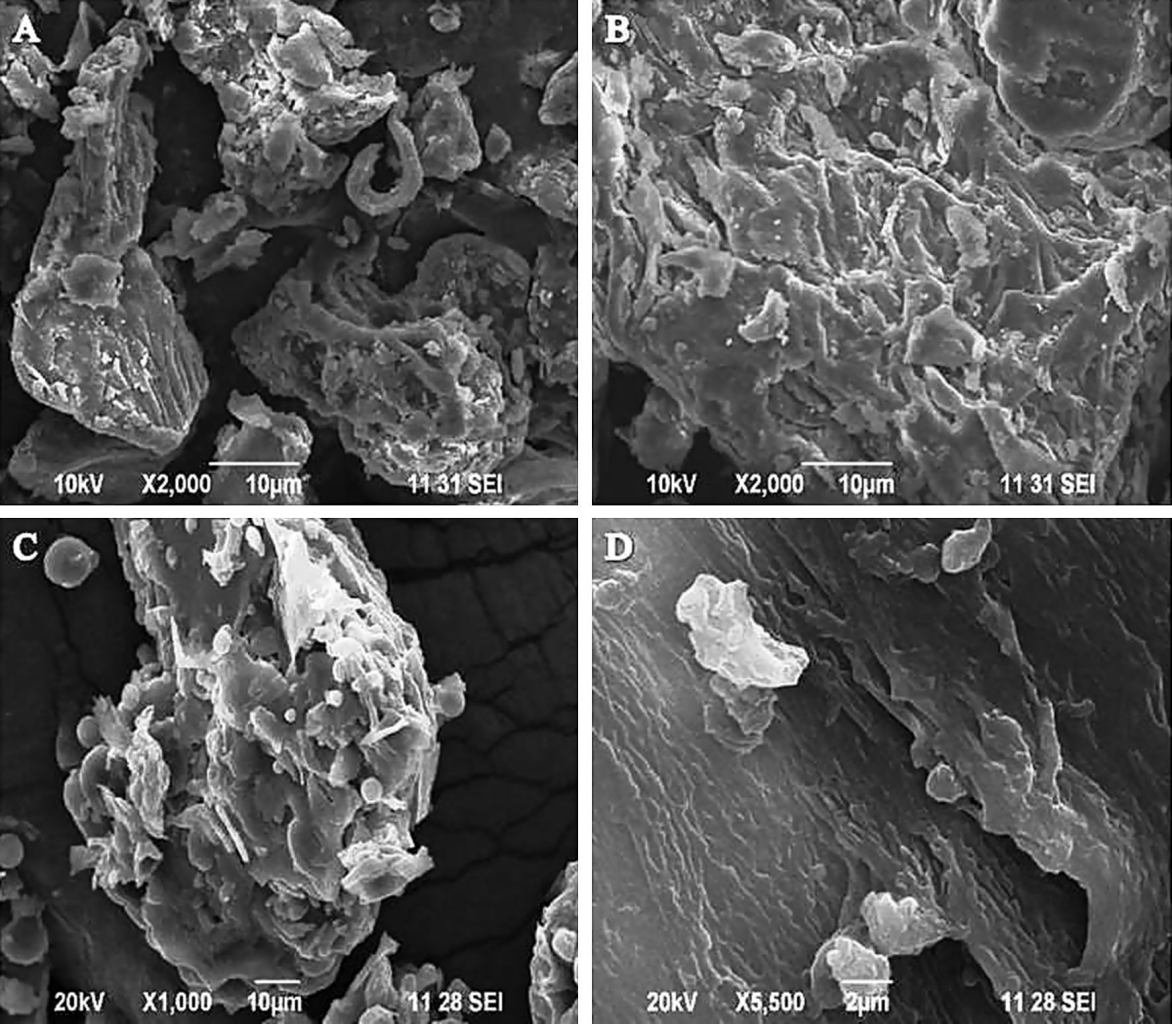

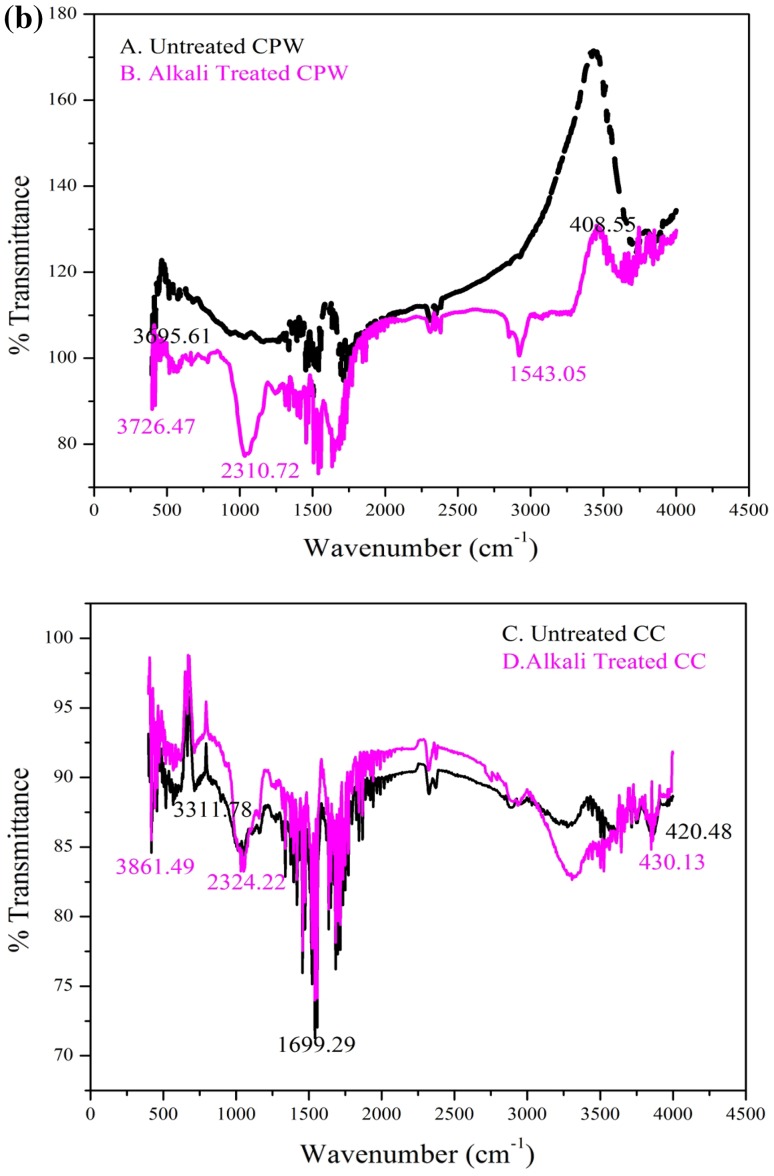
**a** Scanning electron microscopy (SEM) of the coffee pulp waste (CPW) surface. *A* Untreated CPW; *B* pre-treated with NaOH, corncob (CC) surface. *C* untreated CC; *D* pre-treated with NaOH, **b** FTIR of the coffee pulp waste (CPW). *A* untreated CPW; *B* pre-treated with NaOH, corncob (CC); *C* untreated CC; *D* pre-treated with NaOH

The SEM analysis clearly indicated that the NaOH pre-treatment modified the outer layer of the substrates CPW and CC (Fig. 2a). The untreated substrates (CPW and CC) showed a smooth, rigid and highly ordered structure, whereas pre-treatment induced changes in the structure. The pre-treated substrates were uneven, rough and more porous on the surface. These findings indicate that pre-treatment promoted the removal of some external fibers. The removal of the external fibers leads to an increase in the external surface area and the porosity of the substrates. The same changes have been reported after the rice straw was pre-treated by aqueous ammonia (Ko et al. 2009).

### Characterization of alkali-treated substrates

FTIR was used to demonstrate the physical structure and changes in functional groups of CPW and CC before and after pre-treatment (Fig. 2b). FTIR spectroscopy analysis showed obvious changes in the functional groups during the pre-treatment. The chemical bonds of the substrates were investigated in the region of 400–4000 cm^−1^. The pre-treated CPW peaks were located at 3695.61 cm^−1^ and 3726; CC peaks were located at 3311.78 cm^−1^ and 3861.49, respectively. The peaks located at 3726 cm^−1^ and 3861 cm^−1^ correspond to –OH stretching and –CH_2_ stretching, respectively (Sun et al. 2007).

### Optimization of protease production using BBD

The application of modern statistical models to optimize the physicochemical components of the cultural conditions has been increased in current industrial biotechnology because of its universal applicability and suitability. The BBD was applied to identify the optimal conditions for the enhanced production of the protease enzyme. The experimental design is presented in Table 1. The ANOVA of the quadratic regression model (Table 2) exhibits that it was a highly significant model, as evident from the Fisher’s *F* test with a very low probability value (*F *value = 15.67). Values of ‘Prob > *F*’ (0.0001) indicate that the term of the model was significant. The model *F* value of 15.67 implies that the model was significant. There was only a 0.01 % chance that a model *F* value could occur due to noise. The predicted *R* ^2^ (0.6559) and adjusted *R* ^2^ (0.8801) values for protease production were in reasonable agreement with the value of *R* ^2^ (0.9400), which is closer to 1.0, indicating the better fitness of the model in the experimental data. The model for protease production by SSF, three different tests, namely, sequential model sum of squares, lack of fit tests and model summary statistics, were carried out in the present study.

**Table 1:** Box–Behnken design for the variables and the experimentally observed responses

Run	pH	Incubation time	Coffee pulp waste (%)	Corncobs (%)	Protease (U/mL)
1	10	60	3.00	3.00	452
2	6	96	3.00	2.00	456
3	8	60	1.00	1.00	670
4	8	60	5.00	3.00	780
5	8	60	3.00	2.00	914
6	6	60	1.00	2.00	510
7	8	24	3.00	3.00	530
8	8	60	5.00	1.00	487
9	8	24	1.00	2.00	535
10	8	96	1.00	2.00	625
11	8	96	3.00	1.00	513
12	10	60	1.00	2.00	475
13	6	60	3.00	3.00	445
14	6	60	5.00	2.00	460
15	8	96	5.00	2.00	745
16	8	96	3.00	3.00	750
17	8	60	1.00	3.00	655
18	8	60	3.00	2.00	910
19	6	24	3.00	2.00	440
20	10	96	3.00	2.00	445
21	8	60	3.00	2.00	900
22	6	60	3.00	1.00	537
23	8	60	3.00	2.00	920
24	10	60	3.00	1.00	560
25	8	60	3.00	2.00	918
26	8	24	5.00	2.00	600
27	10	60	5.00	2.00	475
28	10	24	3.00	2.00	435
29	8	24	3.00	1.00	615

**Table 2:** Analysis of variance (ANOVA) for the response surface quadratic model

Source	Sum of squares	Df	Mean square	*F* value	*P* value prob > *F*
Model	7.577E + 005	14	54120.14	15.67	<0.0001
*A*––pH	3.00	1	3.00	8.688E − 004	0.9769
*B–*–incubation time	11970.08	1	11970.08	3.47	0.0837
*C*—CPW	494.08	1	494.08	0.14	0.7109
*D*—CC	4408.33	1	4408.33	1.28	0.2775
*AB*	9.00	1	9.00	2.607E − 003	0.9600
*AC*	625.00	1	625.00	0.18	0.6770
*AD*	64.00	1	64.00	0.019	0.8936
*BC*	756.25	1	756.25	0.22	0.6470
*BD*	25921.00	1	25921.00	7.51	0.0160
*CD*	23716.00	1	23716.00	6.87	0.0201
*A* ^2^	5.635E + 005	1	5.635E + 005	163.20	<0.0001
*B* ^2^	1.872E + 005	1	1.872E + 005	54.21	<0.0001
*C* ^2^	1.077E + 005	1	1.077E + 005	31.20	<0.0001
*D* ^2^	1.126E + 005	1	1.126E + 005	32.60	<0.0001
Residual	48340.28	14	3452.88	–	–
Lack of fit	48089.08	10	4808.91	76.57	0.0004
Pure error	251.20	4	62.80	–	–
Cor total	8.060E + 005	28	–	–	–

**Fig. 3:**
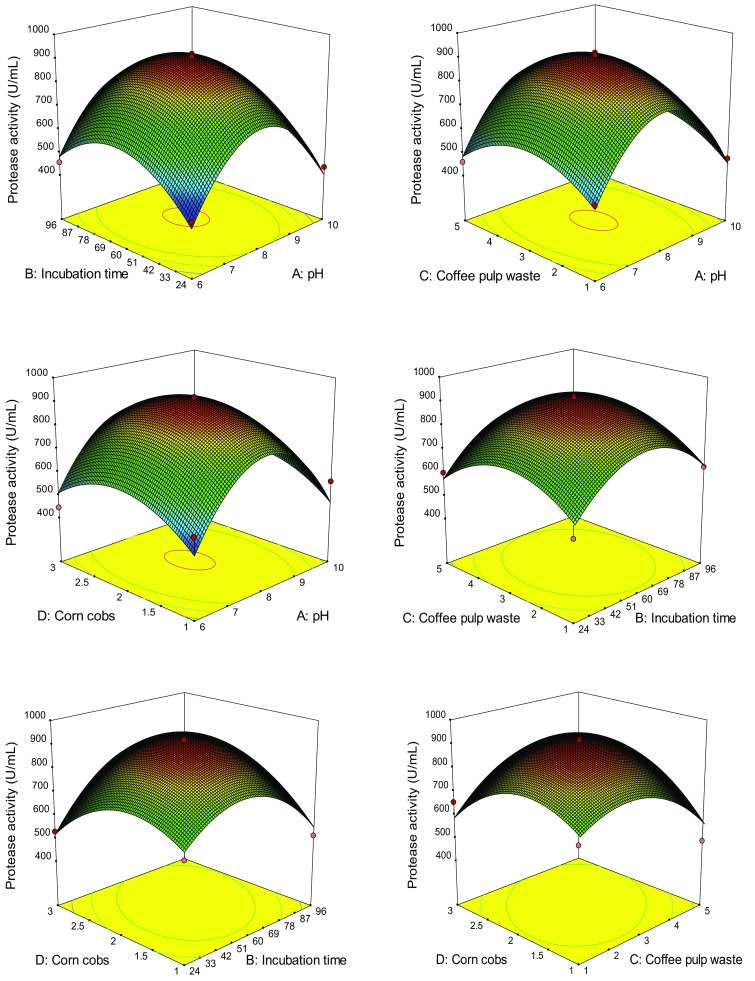
3-D plots of the combined effects of two variables on protease production by *Bacillus* sp. BT MASC 3

The 3-D plot graphical representations were generated (Fig. 3). The results demonstrate that there was a significant relation of pH, incubation time, CPW and CC concentrations for protease production. The optimum levels of the variables were obtained by using BBD. The model predicted a maximum protease activity of 920 U/mL appearing after 60 h cultivation with CPW 3.0 g/L, CC 2.0 g/L of substrate, pH 8 and temperature 37 °C. The results are consistent with previous study reporting the maximum protease (821 U/mL) production by *Bacillus* sp. SKK11 (Govarthanan et al. 2014). The predicted model was validated and experiments were conducted using these optimal conditions. The predicted model values were in good agreement with the values measured in these experiments, thus mitigating the validity of the response model and the necessity for optimal conditions. The graphs highlighted the roles played by the variables for the production of protease.

The coefficients of the regression equation were calculated and the following regression equation was obtained:

2 $$\begin{aligned} Y\; = \;912.40\; - \;0.50A\; + \;31.58B\; + \;6.42C \, + \;19.17D\; - \;1.50AB \, + \;12.50AC\; - \;4.00AD\; + \;13.75BC \, \hfill \\ \quad \; + \;80.50BD\; + \;77.00CD\; - \;294.74A^{2} \; - \;169.87B^{2} \; - \;128.87C^{2} \; - \;131.74D^{2} \hfill \\ \end{aligned}$$

where *Y* stands for protease activity, *A* is pH, *B* is incubation time, *C* is CPW and *D* is CC concentration, respectively. A high degree of similarity of experimental values was observed, thus reflecting the precision and applicability of RSM to optimize the process for protease production. The results are in agreement with the previous studies reporting the significant role of RSM on the enhanced production of secondary metabolites using microorganisms. Optimization of fermentation conditions for the production of protease showed the progress in the rate of production economics and it is also an attractive technology. CPW and CC which are cheap and easily available can be used in SSF for protease production. This indicates the feasibility of the economical production of protease using *Bacillus* sp. MASC 3.

### Determination of molecular weight of protein

**Fig. 4:**
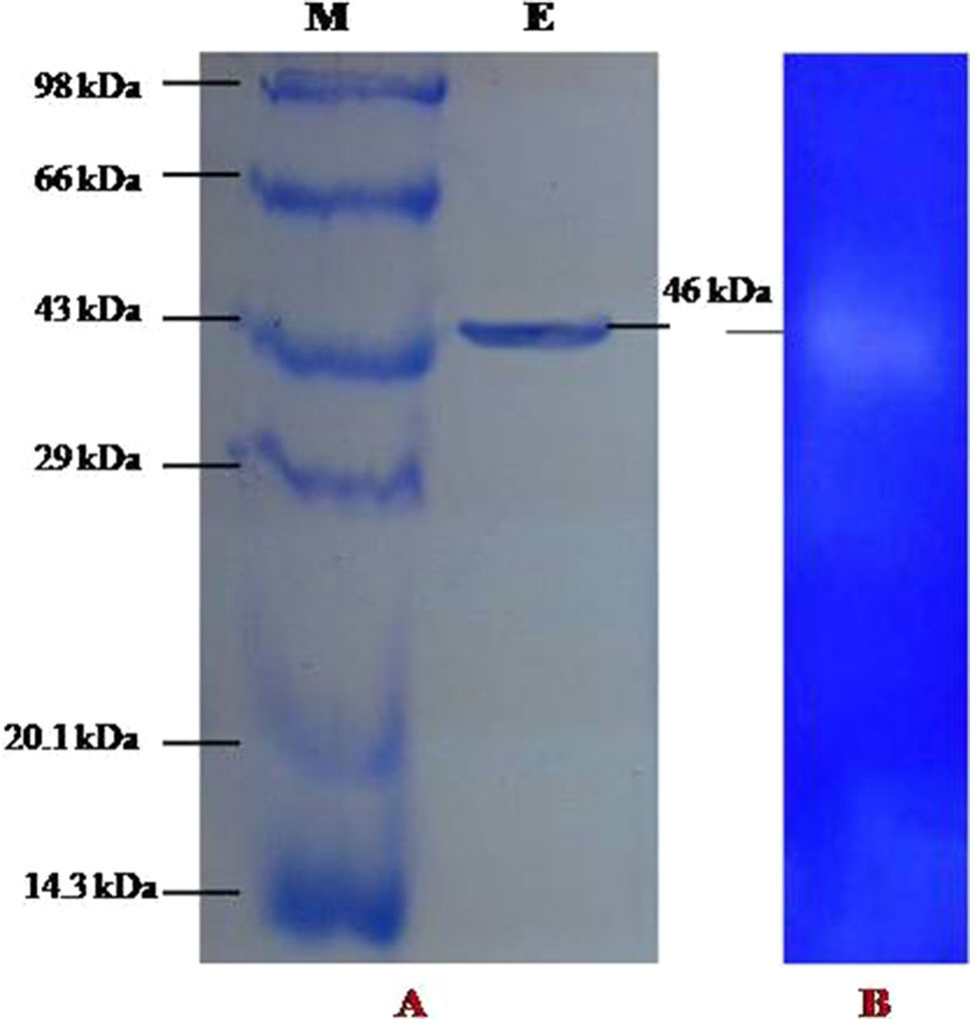
**a** SDS-PAGE, **b** zymogram of purified protease produced by *Bacillus* sp. BT MASC 3, M molecular weight marker (phosphorylase B 97.4 kDa, albumin 66 kDa, ovalbumin 43 kDa, carbonic anhydrase 29 kDa, trypsin inhibitor 20.1 kDa, α-lactalbumin 14.3 kDa), E-purified protease enzyme

SDS-PAGE of the partially purified enzyme from BT MASC 3 showed a single band (Fig. 4a); to confirm it is an enzyme protein band, the protease activity of the purified enzyme was also observed and the apparent molecular weight of the purified protease was 46 kDa. Our results are more or less similar to that of Akel et al. (2009). WHO reported that the purified protease enzyme revealed a molecular mass of 49 kDa. A variety of molecular mass for proteases from other *Bacillus* species had been reported: 75.0 kDa *Bacillus* sp. S17110; (Jung et al. 2007) 30.9 kDa thermophilic *Bacillus* strain HS08. The purified protease enzyme was also subjected to zymogram analysis by SDS-PAGE containing 0.1 % gelatin. The proteolytic activity of the protein is indicated by the clear band (b). Ramakrishna et al. (2010) conducted zymography for protease produced by *Bacillus subtilis* (MTTC N0-10110) on 7 % SDS-PAGE gels containing casein as the substrate at 4 °C.

### Effect of pH and temperature on enzyme activity

**Fig. 5:**
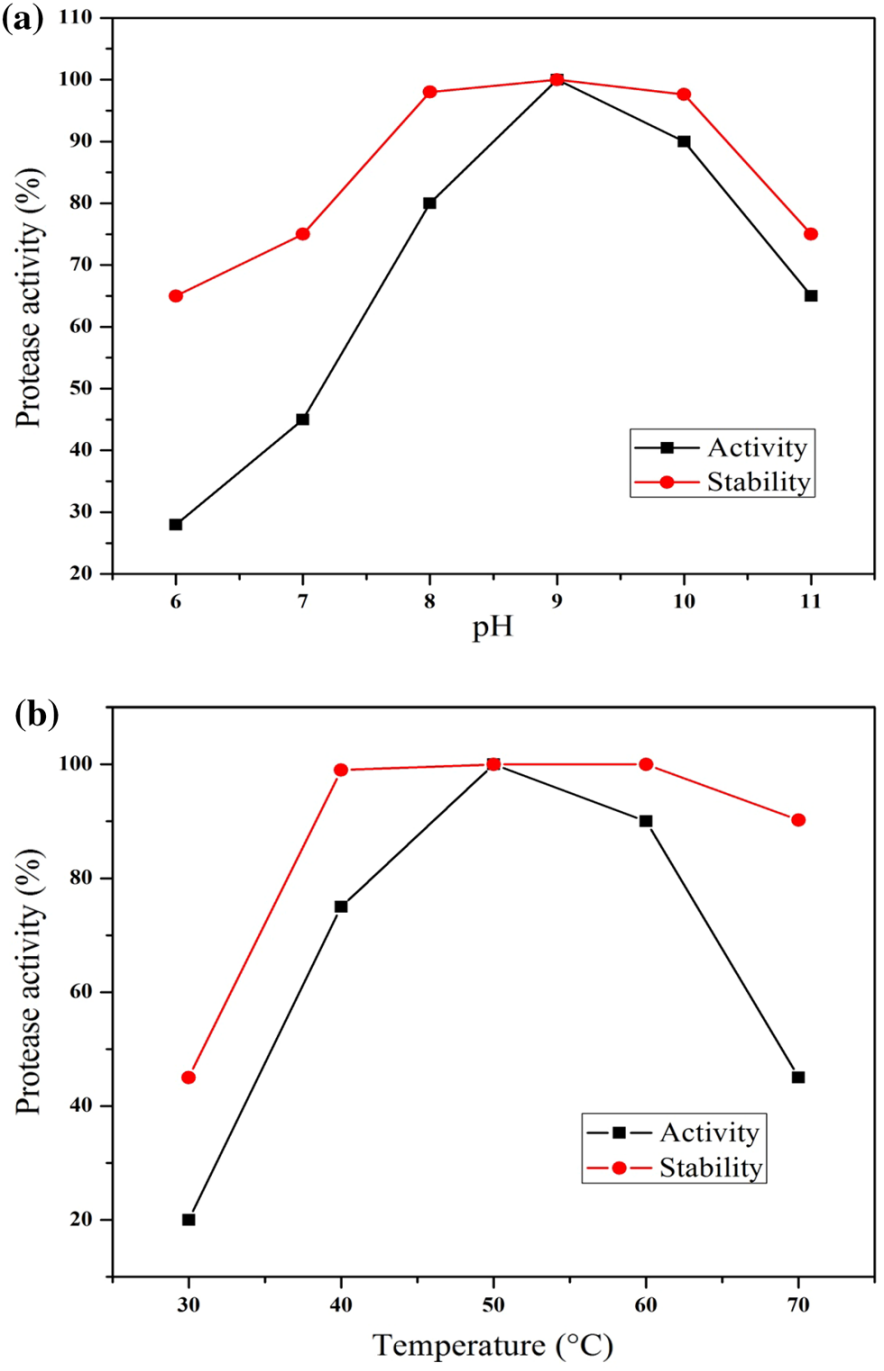
**a** Effect of temperature on the activity and stability of purified protease of *Bacillus* sp. BT MASC 3. **b** Effect of pH on the activity and stability of purified protease of *Bacillus* sp. BT MASC 3

The purified protease was found to be active over a broad range of pH values between 6 and 11 at 37 °C with the maximum pH 9 for hydrolysis of casein. The pH stability of the purified protease showed that the enzyme possessed remarkable stability at pH 8.0–10.0 (97.4–100 %) (Fig. 5a). Based on this observation, BT MASC 3 protease could be classified as an alkaline protease. Similar results were obtained for the optimum pH for enzymatic activity of other *Bacillus* species: pH 7.5 for *Bacillus* sp. S17110, *Bacillus* sp. HS08 (Huang et al. 2006) and *Bacillus subtilis* ITBCCB 148, (Yandri et al. 2008) pH 8.0 for *Bacillus cereus* KCTC 3674, (Kim et al. 2001).

The protease activity of the purified enzyme was measured at temperatures ranging from 30 to 70 °C. The optimum activity of the purified enzyme was exhibited at 50 °C, while only about 10 % of the activity was inactivated after exposure to 60 °C for 10 min. The thermostability of the purified protease showed that it was stable at 40–60 °C for 10 min (Fig. 5b). Previous studies reported that the optimum temperature for production of protease by *B. subtilis* CN2 and *B. pumilus* was at 50 °C *B. subtilis* CN2 and *B. pumilus* was at 50 °C (Aoyama et al. 2000; Uchida et al. 2004). This was supported by Li et al. (1997) who reported that alkaline protease isolated from *Thermomyces lanuginosus* P134 had a broad temperature optimum of 50 °C. Samal et al. (1991) also reported an alkaline protease from *Tritirachium album* Limber to be quite thermostable even up to 50 °C.

## Conclusions

Protease production by *Bacillus* sp. BT MASC 3 under solid-state fermentation was influenced by the physiological and chemical nature of the CPW and CC. The feasibility of using an agro-residue as a possible substrate for protease production was studied using the response surface methodological approach. The optimum conditions for the maximum protease production 920 U/mL using CPW and CC are as follows: pH 8, fermentation time 60 h, with 3.0 g/L of CPW and 2.0 g/L of CC. The enzyme production in this range from this vastly available by-product is significant. The molecular weight of protease enzyme was determined as 46 kDa. The study has demonstrated the suitability of using CPW and CC as a substrate for the production of protease by the statistical optimization process, thereby reducing the cost of protease and could be explored further for waste management through its reuse into value-added products.
